# The occurrence of Enterobacteriaceae producing KPC carbapenemases in a general hospital in Curacao

**DOI:** 10.1186/2047-2994-3-24

**Published:** 2014-08-01

**Authors:** Sandra Erkens-Hulshof, Liane Virginia-Cova, Willemien van Dijk, Juliette Severin, Neil Woodford, Willem Melchers, Patrick Sturm

**Affiliations:** 1 Department of Medical Microbiology, Radboud University Medical Centre, Nijmegen, The Netherlands; 2 Department of Medical Microbiology, Maastricht University Medical Centre, PO Box 5800, Maastricht 6202 AZ, The Netherlands; 3 Analytical Diagnostic Centre, National Laboratory, Willemstad, Curacao, Netherlands Antilles; 4 Department of Hygiene Infection Control, St. Elisabeth Hospital, Willemstad, Curacao, Netherlands Antilles; 5 Department of Medical Microbiology and Infectious Diseases Erasmus MC, University Medical Centre Rotterdam, Rotterdam, The Netherlands; 6 Antimicrobial Resistance and Healthcare Associated Infections Reference Unit, Public Health England, London, UK

**Keywords:** KPC, Carbapenemases, Enterobacteriaceae

## Abstract

**Background:**

Although the presence of Carbapenemase-producing Enterobacteriaceae (CPE) are extensively documented in North and South America. CPE have not been reported from Curacao. However, recently intercontinental spread was suggested of a KPC carbapenemase producing Klebsiella pneumoniae in a patient in the United Kingdom with previous admission to a hospital in Curacao in 2009.

**Findings:**

After the introduction of the CLSI 2010 revised breakpoints, seven patients with carbapenemase-producing Enterobacteriaceae were found in a general hospital in Curacao over a period of 16 months. Four patients carried KPC-2 positive Klebsiella pneumoniae, ST11. Two patients carried KPC-3 positive Klebsiella pneumoniae ST258 and one patient carried a KPC-3 positive Citrobacter freundii. Furthermore, our Klebsiella pneumoniae KPC-2 ST11 strain was matched to the Klebsiella pneumoniae KPC-2 ST11 strain in the United Kingdom.

**Conclusions:**

Introduction of new laboratory methods, and adoption of new guidelines and breakpoints led to the first detection of CPE in Curacao. By matching our Klebsiella pneumoniae KPC-2 ST11 strain to a Klebsiella pneumoniae KPC-2 ST11 strain in the United Kingdom, we suggest that carbapenemase-producing Enterobacteriaceae are probably more prevalent in Curacao than previously recognized.

## Findings

Infections with carbapenemase-producing Enterobacteriaceae (CPE) present clinicians with serious treatment challenges due to limited antibiotic options. They have been associated with high rates of mortality and morbidity, particularly in patients with severe underlying illnesses. The detection of CPE by routine susceptibility testing can be challenging, since the presence of carbapenem resistance genes does not always result in high-level resistance to carbapenems. More and more examples of patients with unrecognized CPE colonization are reported: these patients serve as reservoirs for transmission during health care associated outbreaks, proving the need for rapid and adequate detection of CPE [1].

Several types of acquired carbapenemases have been detected in CPE, of which KPC, VIM, IMP, NDM and OXA-48-like enzymes are most prevalent. Since the detection of the first KPC isolate in 1996 in the United States [2] CPE have become widely distributed throughout the world including South America [3]. Until 2010 no CPE had been reported in Curacao, although intercontinental spread was suggested in a report of a KPC-producing Klebsiella pneumoniae isolated from a patient in the United Kingdom who had travelled to Curacao in 2009 [4]. Here we describe the occurrence of carbapenemase-producing Enterobacteriaceae in Curacao after the introduction of the Clinical and Laboratory Standards Institute (CLSI) 2010 revised breakpoints.

## Methods

St. Elisabeth Hospital in Willemstad, Curacao, is a 355-bed general hospital, providing care to patients from Curacao, and other islands of the Netherlands Antilles. It has fourteen general wards, a 6-bed coronary care unit and a 7-bed intensive care unit. The number of patient beds per room and presence of facilities such as bathroom and toilet differs between first, second and third class rooms. In first class patients have a private room with a private bathroom and toilet. Second class rooms are 2–4 bed rooms with the common use of bathroom and toilet. In third class there are 6–15 beds per room with the common use of bathroom and toilet.

All clinical samples are sent to the Analytic Diagnostic Centre for bacterial culture. Routine identification and susceptibility testing including detection of carbapenemases was according to CLSI standards using VITEK-1 until replacement by a VITEK-2 system in March 2010. With VITEK-2 lower screening breakpoints for the detection of CPE were introduced [5]. Decreased carbapenem susceptibility was confirmed by meropenem E-test and The modified Hodge test was performed.

From March 2010 - July 2011, all isolates with confirmed decreased susceptibility to carbapenems were sent to the Department of Medical Microbiology, Radboud University Medical Centre in the Netherlands. Confirmation of species identification was done by MALDI-TOF. Phenotypic investigations for carbapenemases included inhibition tests with 3-aminophenylboronic acid, clavulanic acid and EDTA. Based on the phenotype PCR for bla_KPC_ genes was performed and amplicons were sequenced. The presence of extended-spectrum beta-lactamase (ESBL) and AmpC genes was investigated by Check-MDR CT101 microarray (Check-Points, Wageningen, The Netherlands). Genotyping of isolates was performed using pulsed-field gel electrophoresis (PFGE) and multi-locus sequence typing (MLST) [6].

A K. pneumoniae strain isolated in the UK and suggestive of intercontinental spread from Curacao to the UK was also investigated [4].

### Ethical considerations

This study is performed on routine clinical bacterial isolates and did not require the agreement of the ethical committee of our institution.

## Results

**Figure 1:**
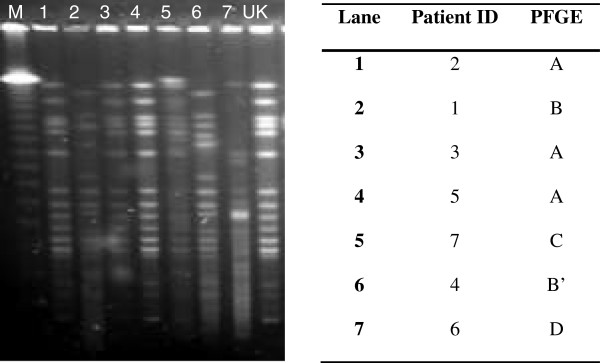
**PFGE profiles of seven KPC producing Enterobacteriaceae found in Curacao and the UK isolate.** M: marker.

In May 2010 the first carbapenemase-producing K. pneumoniae was detected from St. Elisabeth Hospital, Curacao. From May 2010 - July 2011, a total of seven clinical isolates were identified as CPE: six K. pneumoniae and one Citrobacter freundii. Both bla_KPC-2_ and bla_KPC-3_ genes were detected by PCR and sequencing (Table 1). Meropenem MICs ranged from 1 to 32 mg/L. PFGE genotyping revealed an oligoclonal population of CPE (Figure 1). Four K. pneumoniae isolates produced KPC-2 carbapenemase, belonged to ST11 and also produced a CTX-M group 1 ESBL. Two K. pneumoniae isolates with KPC-3 enzymes belonged to the international ST258 lineage, one of which also produced an SHV-type ESBL. Besides its chromosomal AmpC, the C. freundii isolate produced KPC-2 carbapenemase, but did not harbour any additional ESBL genes.

**Table 1:** Details of patients from whom carbapenemase-producing Enterobacteriaceae was isolated

**Patient ID**	**Gender**	**Age (years)**	**Date of positive culture**	**Culture site**	**Species**	**Ward**	**Class**	**KPC**	**PFGE**	**MLST**	**MIC Meropenem**	**Prior history of hospital admission**
**1**	M	58	6/5/10	Rectal surveillance	K. pneumoniae	A	3	3	B	258	1	Colombia
**2**	M	73	13/7/10	Urine	K. pneumoniae	B	3	2	A	11	32	-
**3**	M	15	24/8/10	Urine	K. pneumoniae	C	1	2	A	11	2	-
**4**	M	77	26/10/10	Urine	K. pneumoniae	B	3	3	B’	258	1	Ward A May 2010
**5**	M	78	9/12/10	Rectal surveillance	K. pneumoniae	D	3	2	A	11	4	-
**6**	M	68	17/1/11	Wound	C. freundii	B	3	3	D	ND	1	Ward B Oct. 2010
**7**	M	64	18/6/11	Wound	K. pneumoniae	E	2	2	C	11	8	-

ND: not determined.

The PFGE patterns of the four KPC-2 producing K. pneumoniae isolates were indistinguishable and were identical to that of the K. pneumoniae isolate from the UK, which also produced KPC-2 and belonged to ST11 (Figure 1) [4].

### Patient information

Five of seven carbapenemase producing Enterobacteriaceae were found on third class wards A, B and D, which are on the ground floor of the hospital. Ward A and B are localized in the west wing of the hospital. Ward D is in the east wing of the hospital.

The first K. pneumoniae KPC-3 ST258 discovered in May, was found in a patient admitted to ward A. This patient had been transferred from a hospital in Colombia. In October an identical strain was isolated from a patient in ward B, but who had previously been admitted to ward A in May (Table 1).

Moreover, the patient with KPC-3 C. freundii had been admitted to ward B at the time of positive culture of the second patient with KPC-3 K. pneumonia in October. This patient was screened by the department of Hygiene Infection Control but found negative at the time.

There was no clustering in the hospital or time between the four patients with ST11 K. pneumoniae.

## Discussion and conclusion

Dissemination of CPE has been described in several different countries in South America [7-9]. Results of this study show that CPE are present among clinical isolates in Curacao as well. Until then, no CPE had been detected on the island. However, in March 2010 a new VITEK-2 system was introduced with the CLSI 2010 revised breakpoints for Enterobacteriaceae, including the reduced breakpoints for carbapenem antibiotics. It seems likely that carbapenemase-producing isolates have been present, but unrecognized before this update. This is supported by the report of Virgincar et al. who discussed the nosocomial transmission of a KPC-2 producing K. pneumoniae ST11 strain in the UK in 2009, and which was probably introduced by a patient who had just been discharged from a hospital in Curacao [4].

In our study, the first patient with a KPC-3 producing K. pneumoniae ST258 had recently been hospitalized in a hospital in Colombia, where an outbreak with similar organisms was described 2008 [10]. Since aggressive infection control has been shown to be effective in controlling dissemination of CPE, affected patients in our hospital were placed in contact isolation in single rooms, wherever possible. Strict compliance to hand hygiene for health care workers and family was advised by the Department of Hygiene Infection Control. However, nosocomial transmission may have occurred since the second patient with KPC-3 K. pneumoniae ST258 had been in the same ward as the first patient. Moreover, a third patient with KPC-3 producing C. freundii had been previously admitted to the same ward as the second patient with KPC-3 K. pneumonia, which may indicate plasmid transfer between species as well as undetected KPC producers in one of the two or in both patients.

We have shown that the introduction of new laboratory methods, and adoption of new guidelines and breakpoints led to the first detection of CPE in Curacao. We suggest that carbapenemase-producing Enterobacteriaceae are possibly more prevalent in Curacao than previously recognized.

## Abbreviations

CPE: Carbapenemase-producing Enterobacteriaceae; CLSI: Clinical and Laboratory Standards Institute; ESBL: Extended-spectrum beta-lactamase; PFGE: Pulsed-field gel electrophoresis; MLST: Multi-locus sequence typing.

## Competing interests

All authors report no conflicts of interest relevant to this manuscript.

## Authors’ contributions

PS and SE were responsible for the acquisition and analysis of microbiological data, WD provided patient information and additional hygiene infection control measures. SE drafted the manuscript. JS provided and analyzed MLST data. WM provided and analyzed PFGE data. All authors read, reviewed and provided feedback on the final manuscript.
